# Gene expression profiles and bioinformatics analysis in lung samples from ovalbumin-induced asthmatic mice

**DOI:** 10.1186/s12890-023-02306-w

**Published:** 2023-02-02

**Authors:** Yilan Song, Jingzhi Jiang, Qiaoyun Bai, Siqi Liu, Yalin Zhang, Chang Xu, Hongmei Piao, Liangchang Li, Guanghai Yan

**Affiliations:** 1 Jilin Key Laboratory for Immune and Targeting Research On Common Allergic Diseases, Yanbian University, Yanji, 133002 People’s Republic of China; 2 Department of Anatomy, Histology and Embryology, Yanbian University Medical College, No. 977, Gongyuan Road, Yanji, 133002 Jilin Province People’s Republic of China; 3 Postdoctoral Programme, Research Center, Affiliated Hospital of Yanbian University, Yanji, 133000 People’s Republic of China; 4 Department of Otorhinolaryngology, Affiliated Hospital of Yanbian University, Yanji, 133000 People’s Republic of China; 5 Department of Respiratory Medicine, Affiliated Hospital of Yanbian University, Yanji, 133000 People’s Republic of China

**Keywords:** Microarray, Bioinformatics, lncRNA, mRNA, Asthma

## Abstract

**Background:**

Asthma is characterized by chronic inflammation and airway remodeling. However, limited study is conducted on the gene expression profiles of ovalbumin (OVA) induced asthma in mice. Here, we explored the gene expression profiles in lung tissues from mice with OVA-induced asthma using microarray and bioinformatics analysis.

**Methods:**

For establishment of OVA-induced asthma model, mice first received intraperitoneal sensitization with OVA on day 0, 7 and 14, followed by atomizing inhalation of OVA 3 times a week for 8 weeks. The lung tissues were collected and subjected to microarray analysis, bioinformatics analysis and expression validation.

**Results:**

Microarray data of lung tissues suggested that 3754 lncRNAs and 2976 mRNAs were differentially expressed in lung tissues between control and asthmatic mice, including 1647 up-regulated and 2106 down-regulated lncRNAs, and 1201 up-regulated and 1766 down-regulated mRNAs. GO analysis displayed that the up-regulated genes were enriched in inflammatory response, leukocyte migration involved in inflammatory response, and Notch signaling pathway. KEGG pathway analysis indicated that the enriched pathway terms of the up-regulated gene included Toll-like receptor signaling pathway and Th17 cell differentiation signaling pathway. Additionally, based on the previously published literatures on asthma and inflammation, we screened out down-regulated genes, such as Smg7, Sumo2, and Stat5a, and up-regulated genes, such as Myl9, Fos and Tlr4. According to the mRNA-lncRNA co-expression network, we selected lncRNAs associated with above genes, including the down-regulated lncRNAs of NONMMUT032848, NONMMUT008873, NONMMUT009478, and NONMMUT006807, and the up-regulated lncRNAs of NONMMUT052633, NONMMUT05340 and NONMMUT042325. The expression changes of the above genes were validated in lung tissues by real-time quantitaive PCR and Western blot.

**Conclusions:**

Overall, we performed gene microarray on lung samples from OVA-induced asthmatic mice and summarized core mRNAs and their related lncRNAs. This study may provide evidence for further research on the therapeutic targets of asthma.

**Supplementary Information:**

The online version contains supplementary material available at 10.1186/s12890-023-02306-w.

## Background

Bronchial asthma is a common and frequently-occurring disease of the respiratory system, characterized by airway inflammation and airway hyperreactivity (AHR) [1]. It is generally accepted that chronic airway inflammation is one of the important mechanisms leading to AHR, and airway remodeling is the pathological basis causing irreversible AHR and airway obstruction. Ovalbumin (OVA) is one of the main asthmatic allergens. It is commonly used to establish asthma models in animals, models of airway inflammation and remodeling in, which is characterized by airway inflammation, intra-epithelial eosinophil infiltration, TH2 immune response, airway remodeling (such as goblet cell hyperplasia, airway smooth muscle cell (ASMC) hypertrophy and/or hyperplasia, basal membrane thickening, subepithelial fibrosis, bronchial angiogenesis, and collagen deposition), and AHR [2]. However, despite treatment following guidelines, patients with asthma still undergo exacerbations, which may finally lead to the higher disease morbidity and mortality [3, 4]. Overall, asthma remains an important health care problem and a significant financial burden to patients and society. Hence, there is an urgent need to develop better strategies and identify new therapeutic targets for asthma.

Long non-coding RNAs (lncRNAs) are non-protein-coding RNAs with the length of > 200 nt [5]. They can play functions of epigenetic regulation, act as a sequence-specific tether for protein complexes, specify subcellular compartments or localization, and further regulate development, differentiation and disease pathogenesis [6]. Recently, lncRNAs are reported to exert regulatory roles in the airway inflammation of asthmatic subjects [7]. LncRNA BCYRN1 could facilitate the proliferation and migration of ASMCs of asthmatic rat through up-regulation of transient receptor potential [8]. Down-regulating lncRNA-AK149641 in OVA-induced asthmatic mice could alleviate airway inflammation, mucus secretions, decrease expression of interleukin-6 and tumor necrosis factor alpha [9]. LncRNA Malat1 could inhibit the platelet-derived growth factor BB mediated ASMCs proliferation and migration through miR-150-eIF4E/Akt Signaling in the airway remodeling in asthma [10]. Besides, the expression profiles of lncRNAs in CD8 positive T cells were different in different stages of proliferation, demonstrating the association with peripheral CD8 positive T cells in severe asthma [11]. Qiu et al. [12] reported that the lncRNA MEG3 regulated Treg/Th17 balance via targeting miR-17/RORγt in the asthma patients.

At present, microarray analysis on asthma pathologies is emerging. Chen et al. [7] indicated that through the microarray and RNA-sequencing conducted on healthy donor and patients with severe asthma, miR-133a-3p-*EFHD2/CNN2*-AC144831.1 interactions and miR-3613-3p-*CD44/BCL11B*-LINC00158/CTA-217C2.1/AC010976.2/RP11-641A6.2 interactions were speculated to involve with the development of severe asthma. Kim et al. [13] demonstrated four interconnected hub genes, FOXO1, RUNX1, SP1 and APP from DNA methylation and gene expression networks in lung tissues of OVA-induced asthma model (15 days) of mice. He et al. [14] performed microarray analysis on endobronchial epithelial brushing samples from asthma patients and conducted validation experiments using house dust mite (HDM)-(14 days) and OVA-induced (26 days) allergic inflammatory asthma model. They suggested that ANO7, PYCR1 and UBE2C might play a central roles in the HDM- and OVA-induced allergic inflammatory asthma. Zhang et al. [9] performed gene microarray analysis on lung tissues of OVA-induced asthmatic mice (30 days), and indicated that the lncRNA-AK149641 may serve as a promising target for the treatment of asthma. However, the microarray analysis on lung tissues from the OVA-induced chronic airway remodeling (8 weeks) mouse model is lacking.

Herein, the purpose of this work was to determine the differentially expressed mRNAs and lncRNAs in lung tissues of OVA-induced asthmatic mice. Gene microarray and bioinformatics analysis, including Gene Ontology (GO) analysis, the Kyoto Encyclopedia of Genes and Genomes (KEGG) database analysis, and co-expression network analysis of lncRNA and mRNA, were performed. Our findings may provide evidence on differentially expressed mRNAs and lncRNAs for asthma.

## Methods

### Animals

Female BALB/c mice (4–8 week; weight 20–22 g; n = 32) were obtained from Experimental Animal Center of Yanbian University (Yanji, China). All mice were housed in the specific pathogen-free conditions with 22 ± 2 °C of room temperature and 50–60% of relative humidity. All methods were performed in accordance with the relevant guidelines and regulations. The experimental protocol was approved by the Ethics Committee of Science and Technology Department of Jilin Province (SYXK (JI) 2020-0009). The study is reported in accordance with ARRIVE guidelines.

### Establishment of OVA induced asthma model00

Mice were divided into control and asthmatic group (n = 11 per group). To construct the asthmatic model, OVA (10 µg, Sigma-Aldrich, USA) and aluminium hydroxide adjuvant (1 mg, InvivoGen, USA) were dissolved in 200 µL of sterile PBS, and then intraperitoneally (i.p.) injected on day 0, 7 and 14 for sensitization. In control group, mice were injected with equal volume of PBS. After 3 days after sensitization, the mice were excited by ultrasonic atomizing inhalation with 5% OVA for 30 min and 3 times a week for 8 weeks [2]. During excitation, the mice were under asthmatic attack and showed some symptoms of agitation, cyanosis, tachypnea and bucking. At 48 h after the final inhalation, mice were sacrificed using 100 mg/kg of pentobarbital sodium.

### Sample collection

At 48 h after the final inhalation, mice were sacrificed using 100 mg/kg of pentobarbital sodium. The bronchoalveolar lavage fluid was collected to analyze cytokine levels. Then, the left and right lung tissues were collected. The left lung tissues were subjected to HE staining and Masson staining. The right lung tissues from 3 mice of each group were used for gene microarray analysis and those from 8 mice of each group were used for validation of gene and protein expressions.

### Verification of asthma model establishment

The asthma model establishment was verified by HE and Masson staining of left lung tissues, as well as cytokine levels (IL-4, IL-5, IL-13 and IFN-γ) in bronchoalveolar lavage fluid (n = 5). Lung tissues were fixed, dehydrated, embedded in paraffin, and cut into 4 μm sections. The lung sections were stained with HE staining kit (#G1120, Solarbio, Beijing, China) and Masson staining kit (#G1345, Solarbio, Beijing, China), according to the instructions. Levels of IL-4, IL-5, IL-13, and IFN-γ were measured with corresponding ELISA Kits (#D4050, M5000, D1300B, and QK285; R&D Systems, Minneapolis, MN, USA), according to the manufacturer’s instructions.

### Quantitative real-time PCR (qRT-PCR)

Total RNAs from the right lung tissues were extracted with RNA isolation kit (Tiangen, DP451, Beijing, China). The cDNA samples were obtained by reverse transcription with Reverse Transcription kit (Tiangen, KR118-02). The qRT-PCR was performed with SYBR Green qRT-PCR kit (Tiangen, KR123). The primer sequences for mRNA and lncRNAs were listed in Table 1. Expression levels were calculated by methods of 2^–△△Ct^, and all values were normalized to *GAPDH*.

**Table 1:** Primer sequences of mRNAs and lncRNAs for qRT-PCR

Genes	Forward	Reverse
*mRNA*		
*Fos*	GGGACAGCCTTTCCTACTACC	GATCTGCGCAAAAGTCCTGT
*Smg7*	GCAAGGCCAAGCCAAGAATC	TCACCAGCTCCCACTATGGA
*Cpsf6*	TAATGGCGATGCCCCAGAAG	ATCCAACACCAACAAGGGCA
*Cdk1*	AAGCGAGGAAGAAGGAGTGC	GGCCGAAATCAGCCAGTTTG
*Sumo2*	CCGACGAGAAACCCAAGGAA	TCAGGATGTGGTGGGACCAA
*Cdc20*	GCACTCACTGCTTCAACTGG	ATTCTGAGGTTTGCCGCTGA
*Stat5a*	CAGCATTTCCCCATCGAGGT	AAGGTGCTTCTGGGACATGG
*Stat5b*	CCCAACATTGGGGAAAGGGT	GGGCAGAAGTCCATAGTAGCC
*Myl9*	TTGACAAGGAGGACCTGCAC	CCGGTACATCTCGTCCACTT
*Tlr4*	CGCTGCCACCAGTTACAGAT	AGAGGTGGTGTAAGCCATGC
*Fxyd2*	GCATCTGACAGCTTCCCCTT	AGTTCCTGGGTACCACCTGT
*Topbp1*	ACAGATGAGGAAGCATTGAAGA	GCTCCCAACTTCTCCTGCAA
*GAPDH*	TGCACCACCAACTGCTTAGC	GGCATGGACTGTGGTCATGAG
*lncRNA*		
NONMMUT032848	TCAGAAGCCTCAGGTACGGA	AATGTGAATTGCTGGCCTTAGT
NONMMUT008873	AGAAGGCTGCTTCTTTGACTGTC	TTTAGGACACAGGGTCTTGCTAC
NONMMUT009478	GCATTTGCTACCACATCTGAGTT	GATGGACAGTACAATTTATGGGAGT
NONMMUT052633	TCTGGACTCTTCTATGGACCTTTG	CATTAACTGACACGGTTGTGATTCT
NONMMUT006807	GTGGAAACCAGAGGTAGACATTG	GGCTCAGAGAATCAGAGAGCAC
NONMMUT053402	CACCTGAGCAGTATGATGGTAACA	TAGACCTCAAGATAAGCAGCAATAG
NONMMUT042325	GCAGATGCCTCTCCCAGTTAG	GTGATGCTTGTTCAAGTCGTCA

### Western blot

For extraction of total proteins, the right lung tissues were homogenized using Automatic sample crusher (Tissuelyser II, Qiagen, Germany), and subjected to lysis with RIPA and Protease Inhibitor K (Beyotime, Jiangsu, China). Proteins (20 µg per lane) were separated on 12% SDS-PAGE, and transferred onto PVDF membrane with semi-dry transfer. After blocking with 5% non-fat milk (#1172GR500, BioFroxx, Germany) for 2 h, the membrane was successively incubated with primary antibodies overnight at 4 °C, and secondary antibodies for 1 h at room temperature. Primary antibodies were as follows: Fos antibody (#222699, 1:1000, abcam), SMG7 (suppressor with morphological defects in genitalia 7) (#254610, 1:1000, abcam), Sumo2 (small ubiquitin-like modifier 2) (#233222, 1:1000, abcam), Stat5a (Signal transducer and activator of transcription 5a) (#32043, 1:1000, abcam), Myl9 (myosin regulatory light chain 9) (#191393, 1:1000, abcam), and TLR4 (Toll-like receptors 4) (#13867, 1:1000, abcam). Secondary antibody was Goat Anti-Rabbit IgG H&L (HRP) (#7090, 1:5000, abcam).

### Microarray analysis

Microarray analysis was performed according to the Affymetrix manufacture’s protocol. Briefly, obtained cDNAs were hybridized with GeneChip® Mouse Transcriptome Array 1.0 (44,699 genes; 22,829 lnc RNA) for 16 h at 45 °C. After washing and staining by Affymetrix Fluidics Station 450 (Affymetrix, California, USA), the gene chips were scanned using GeneChip® Scanner 3000 7G (eBioscince, California, USA). Data were analyzed using Affymetrix default analysis settings by Robust Multichip Analysis algorithm. Data was filtered by *p* value of < 0.05 and fold change of > 1.2.

### Bioinformatics analysis

Differentially expressed genes were identified using the Limma package. The hierarchical Clustering analysis of differentially expressed lncRNAs was analyzed with Cluster_Treeview software from Stanford University (Palo Alto, CA, USA). The relationship of up-regulated genes and down-regulated genes were visualized by scatter plot. Pathway enrichment was performed with the KEGG database (https://www.genome.jp/kegg/) [15]. Additionally, the differentially expressed mRNAs were analyzed by GO database (http://geneontology.org).

A co-expression network of mRNAs and lncRNAs was constructed according to the normalized signal intensity. Briefly, associations between lncRNAs and mRNAs were predicted by calculating the Pearson co-expression coefficient using the R function cor.test. The significant mRNA-lncRNA pairs were screened to construct the co-expression network of mRNAs and lncRNAs, and the correlation coefficient cut off value was 0.99. Cytoscape (vension: 3.6.0) was used for the visualization of the co-expression network. The degree was calculated to represent the centrality of gene or lncRNA in a network.

### Statistical analysis

In the GO and KEGG pathway enrichment analysis, Fisher’s exact test was used to calculate *p* value, and the *p* value was adjusted with Benjamini–Hochberg method to obtain p-adjusted value. Experimental data are represented as mean ± SD of at least three experiments. Significant differences were assessed by Student’s t-test. *p* < 0.05 was considered as statistically significant.

## Results

### Hierarchical clustering analysis of LncRNA and mRNA expression in OVA-induced asthmatic mice

**Fig. 1:**
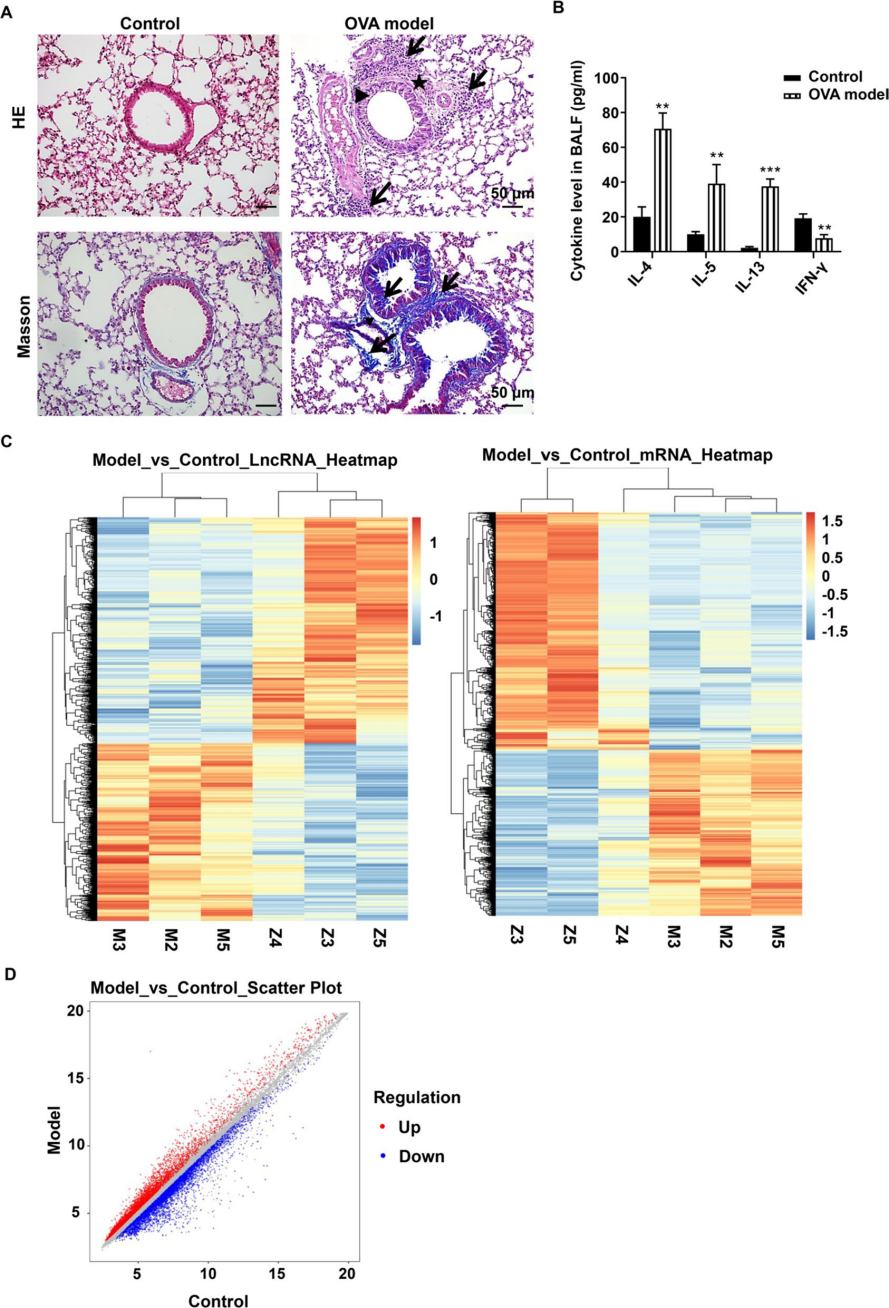
Verification of asthma model establishment and analysis of differentially expressed lncRNAs and mRNAs in lung tissues. Asthma model was established in mice (n = 11). **A** Asthma model establishment was assessed by HE staining and Masson staining. Scale bar = 50 μm. Black arrows indicate inflammatory cell infiltration and collagen deposition. Stars indicate smooth muscle layer, and triangles indicate subepithelial basement membrane, respectively. **B** Asthma model establishment was evaluated by measuring cytokine levels of IL-4, IL-5, IL-13 and IFN-γ in BALF (bronchoalveolar lavage fluid). **C** Hierarchical clustering was performed for differentially expressed lncRNAs and mRNAs in lung tissues (n = 3). Red indicates the up-regulated lncRNAs or mRNAs and blue indicates down-regulated lncRNAs or mRNAs. lncRNA = long noncoding RNA. M indicates model group, Z indicates control group. **D** Scatter plot showing the correlation of mRNAs and lncRNAs between asthma model and control mice

We first assessed asthma model establishment by analyzing the histopathological changes of the lung tissues. HE and Masson staining showed that the OVA group had more inflammatory cell infiltration, thicker smooth muscle layer and subepithelial basement membrane and more collagen deposition around the airway than the control group (Fig. 1 A). Then, we evaluated asthma model establishment by measuring cytokine levels in bronchoalveolar lavage fluid. As shown in Fig. 1B, there were significantly higher levels of TH2-phenotype cytokines (IL-4, IL-5 and IL-13), but significantly lower levels of TH1-phenotype cytokine (IFN-γ) in OVA group than control group (*p* < 0.05). These results indicate the successful establishment of asthma model in mice.

To investigate the differentially expressed lncRNAs and mRNAs in the lungs of control and asthma model mice, we performed microarray and hierarchical clustering analysis. There were approximately 3754 differentially expressed lncRNAs and 2967 differentially expressed mRNAs. Among them, there were 1647 up-regulated lncRNAs and 2106 down-regulated lncRNAs, and 1201 up-regulated mRNAs and 1766 down-regulated mRNAs (fold change > 1.2, *p* < 0.05) (Fig. 1 C). Then, we analyzed the correlation between up-regulated gene and down-regulated genes as shown by the scatterplots (Fig. 1D).

### GO enrichment analysis of differentially expressed genes in OVA-induced asthmatic mice

**Fig. 2:**
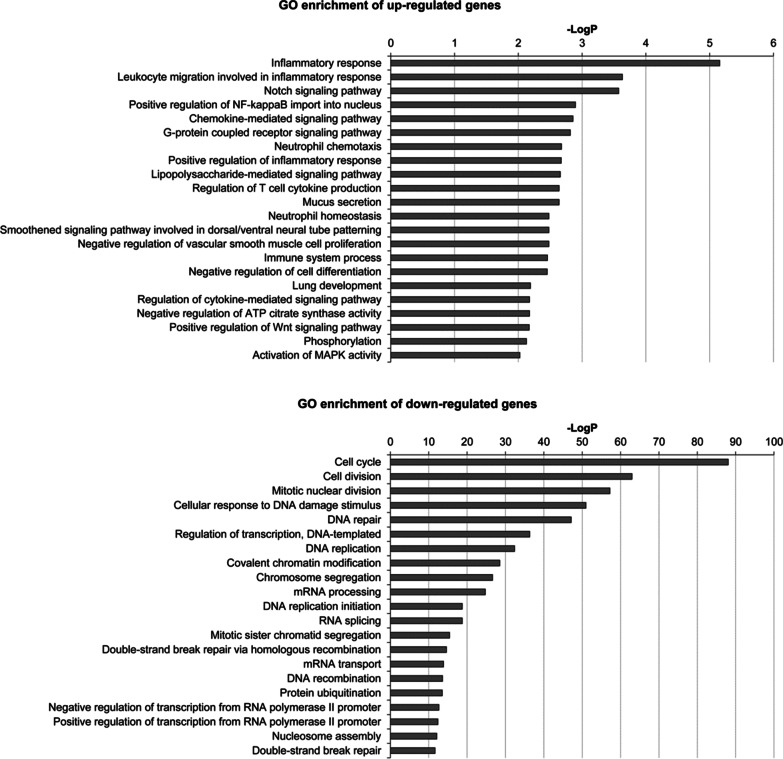
Gene ontology (GO) analysis of differentially expressed mRNAs between control and asthma mice. The top 22 enriched GO terms of up-regulated and down-regulated genes were presented, respectively. The larger − LogP indicates a smaller *p* value. The Y-axis noted the significantly enriched biological process of GO

To explore the functional effects of differentially expressed genes, GO enrichment analysis performed. The −LogP was considered as *p *value of each GO term, and the larger −LogP indicates the smaller *p *value. Resultantly, the up-regulated genes were significantly enriched in 131 GO terms while the down-regulated genes were significantly enriched in 379 GO terms. The top 22 GO terms were illustrated in Fig. 2. The increased GO terms (*p* < 0.05) consisted of inflammatory response, leukocyte migration, Notch signaling, positive regulation of NF-kappaB nuclear translocation and neutrophil chemotaxis, G-protein coupled receptor signaling, neutrophil chemotaxis, response to molecule of bacterial origin, positive regulation of inflammatory response, lipopolysaccharide-mediated signaling pathway, nitric oxide production, regulation of T cell cytokine production, and mucus secretion. The decreased GO terms (*p* < 0.05) included Cell Cycle, Cell Division, Mitotic Nuclear Division, Cellular Response to DNA Damage Stimulus, DNA Repair, Regulation of Transcription, DNA-templated, DNA Replication, Covalent Chromatin Modification, Chromosome Segregation, mRNA Processing, DNA Replication Initiation, RNA Splicing, Mitotic Sister Chromatid Segregation, Double-strand Break Repair Via Homologous Recombination, mRNA Transport, DNA Recombination, Protein Ubiquitination, Negative Regulation of Transcription from RNA Polymerase II Promoter, Positive Regulation of Transcription from RNA Polymerase II Promoter, Nucleosome Assembly, Double-strand Break Repair.

### KEGG pathway enrichment analysis of differentially expressed genes in OVA-induced asthmatic mice

**Fig. 3:**
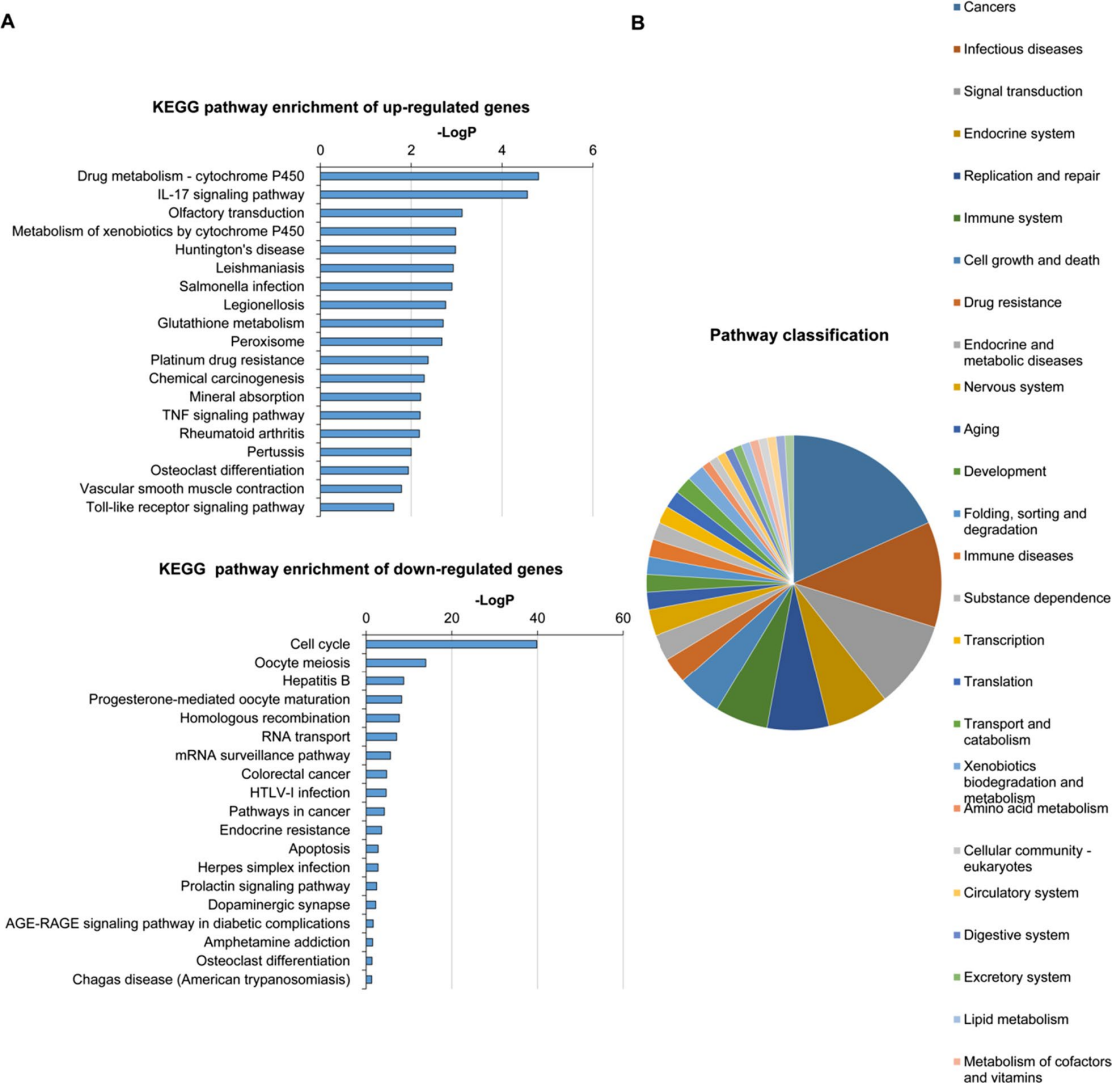
Pathway enrichment analysis of mRNAs between control and asthmatic mice. **A** KEGG pathway analysis was performed. The X-axis represents − LogP, whose size is proportional to *p* value, and the Y-axis represents the significantly enriched pathways. **B** Pie chart of classifications of significant pathways were illustrated

To further understand the key pathways related to these genes, we conducted the pathway analysis using the KEGG database. As shown in Fig. 3A, the KEGG pathway enrichment of up-regulated genes were mainly related to IL-17 signaling pathway, TNF signaling pathway Toll-like receptor signaling pathway, etc. The KEGG pathway of down-regulated genes were mainly enriched in cell cycle, Hepatitis B and so on. Of interest, pathways were mostly involved in cancer, infectious disease, signal transduction and immune system (Fig. 3B). Above all, the results of the GO and KEGG pathway analysis revealed that the differentially expressed mRNAs were mostly involved in the infectious diseases and immune system.

### LncRNA–mRNA co-expression network

**Fig. 4:**
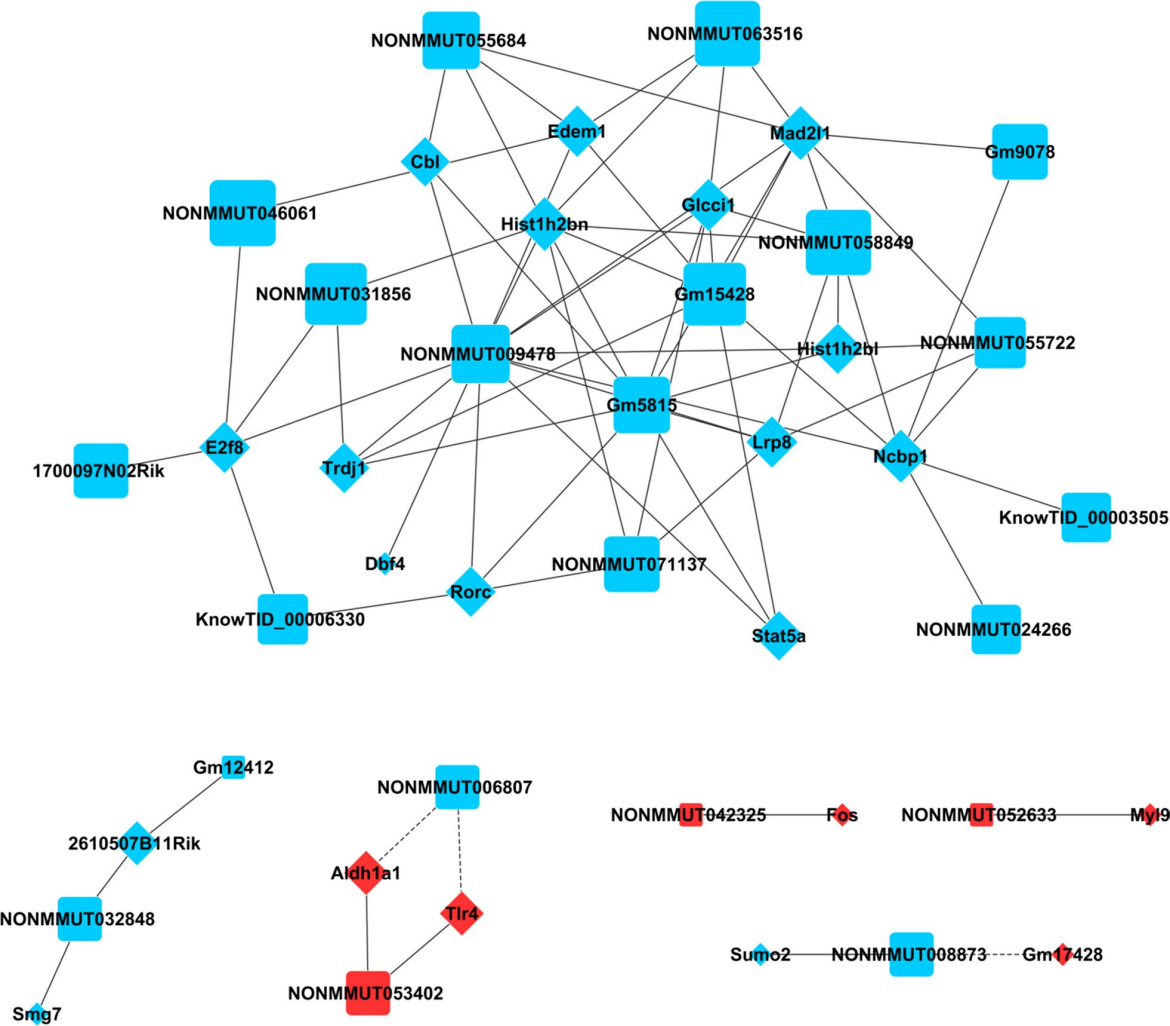
LncRNAs and mRNAs co-expression network. Network of selected lncRNAs and mRNAs. Circle indicate mRNAs and squares indicate lncRNAs. The betweenness centrality was highlighted by the size of the circle. Red and blue denotes up-regulation and down-regulation, respectively. Lines indicate the relationship between the groups. The positive association was indicated by solid line and the negative association was indicated by dotted line

A gene co-expression network was constructed to reveal the associations between lncRNA and mRNA in the control and asthma group. According to the published literatures on asthma and inflammation, 11 mRNAs and their regulatory 17 lncRNAs were screened out to construct the lncRNA-mRNA co-expression network (Fig. 4). LncRNA NONMMUT032848 was positively associated with *Smg7*. NONMMUT008873 was positively associated with *Sumo2*. NONMMUT009478 was positively associated with *Stat5a*. NONMMUT007974 was positively associated with *Cdk1*. LncRNA NONMMUT035972 was positively associated with *Topbp1*. NONMMUT052633 was negatively associated with *Myl9*. NONMMUT053402 was positively associated with *Tlr4*. LncRNA NONMMUT006807 was negatively associated with *Tlr4*. NONMMUT042325 was positively associated with *Fos*. NONMMUT035277 and NONMMUT007470 were positively associated with *Cpsf6*. B630019A10Rik and NONMMUT009675 were positively associated with *Stat5b*. NONMMUT074536, KnowTID_00003505, and NONMMUT074536 were positively associated with *Cdc20*. NONMMUT005281 was positively associated with *Fxyd2* (Fig. 4).

### Validation of genes by qRT-PCR and Western blot analysis

**Fig. 5:**
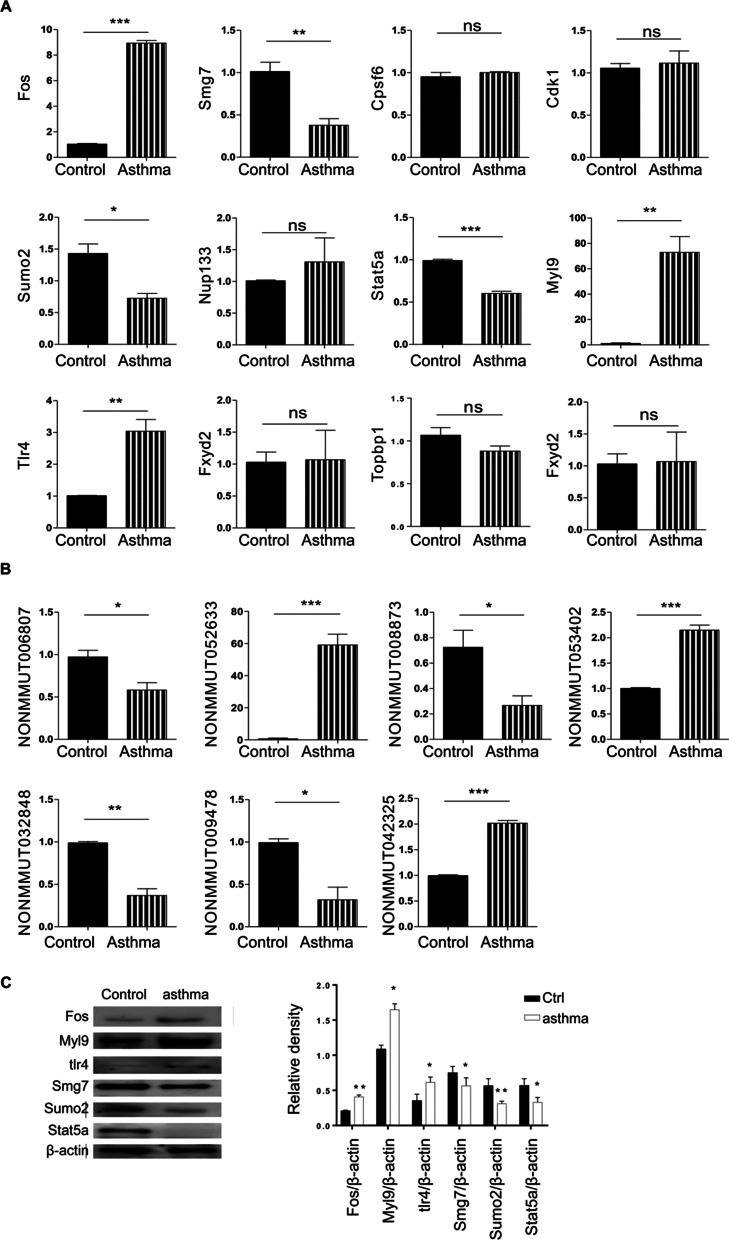
Twelve selected mRNAs and seven selected lncRNAs were validated in vivo. Transcriptional and protein levels of the lncRNAs and mRNAs were determined by qRT-PCR and western blot (n = 8). **A** qRT-PCR results of mRNAs. **B** qRT-PCR results of lncRNAs. **C** Western blot results. The original blots are represented in Additional file 1: Figure S1.*p < 0.05, **p < 0.01, ***p < 0.001 compare to control group. ns, not significant

The lung tissues of each group were extracted for gene and protein validation. Analysis by qR-PCR showed that the expressions of *Smg7, Sumo2*, and *Stat5a* were significantly reduced and those *Myl9, Fos* and *Tlr4* were significantly elevated in the lungs of OVA-induced asthmatic mouse compared to the control group (*p* < 0.05) (Fig. 5A). The expressions of *Cpsf6, Cdc20, Stat5b, Cdk1, Topbp1* and *Fxyd2* were no statistically significant (Fig. 5A). Then, qRT-PCR was used to validate the related lncRNAs of *Smg7, Sumo2, Stat5a*, *Myl9, Fos* and *Tlr4.* As shown in Fig. 5B, the expressions of NONMMUT032848, NONMMUT008873, NONMMUT009478, and NONMMUT006807 were down-regulated in lungs of model mice; in contrast, those of NONMMUT052633, NONMMUT05340 and NONMMUT042325 were significantly up-regulated (*p* < 0.05). Additionally, Western blot was performed to determine the expression of SMG7, SUMO2, and STAT5, Myl9, Fos and Tlr4. Compared with the control group, the expression of Fos, Myl9 and Tlr4 in the asthma model was increased, while the expression of Smg7, Sumo2 and Stat5a was decreased (Fig. 5C).

## Discussion

The asthma model, which is characterized by airway inflammation, can be induced by OVA stimulation for less than 30 days [9]. In this study, to establish asthma model characterized by airway remodeling, we prolonged OVA stimulation to 8 weeks. The prolonged OVA stimulation may aggravate the inflammatory response and lead to persistent inflammation [16]. The pathological changes in the airway epithelium such as subepithelial basement membrane thickening, smooth muscle cell hyperplasia and hypertrophy may occur under the action of cytokines and inflammatory mediators [2]. Thus, both airway inflammation and airway remodeling characteristics were present in the asthma model induced by prolonged OVA stimulation. The pathological findings in this study suggest that the airway remodeling asthma model was successfully established.

After successful asthma model establishment, we determined the differentially expressed mRNAs and lncRNAs in the lung tissues from control and OVA-induced asthmatic mice using a transcriptomics based approach. There were differentially expressed 3754 lncRNAs (1647 up-regulated and 2106 down-regulated lncRNAs) and 2967 mRNAs (1201 up-regulated and 1766 down-regulated mRNAs). Through bioinformatics analysis, we found that the variety of the differentially expressed mRNAs from lung samples of control and model mice were involved in Cell cycle, Apoptosis, and Toll-like receptor signaling pathway, etc., which are mostly associated with the development of asthma. Among them, 6 mRNAs and 7 of their potential regulator lncRNAs were validated by qRT-PCR and/or Western blot. Most of them were enriched in the cancer, infectious disease and signal transduction. These mRNAs and lncRNAs may enrich the potential therapeutic targets of intervention of allergic inflammatory and airway remodeling asthma.

The mRNAs from our microarray data are those widely reported and have important roles in disease control. The small ubiquitin-like modifier (SUMO) can regulate protein post-translational modification, which consists of 3 SUMO paralogs, including *Sumo1, Sumo2 and Sumo3*, and the latter two always form the Sumo2/3 complex [17]. Sumo2/3 is mainly involved in the acute stress response, such as inflammation and hypoxia [18]. Brandsma et al. [19] demonstrated that there was markedly decreased Sumo2 in lung tissues of severe chronic obstructive pulmonary disease (COPD) patients by STRING protein–protein interaction network analysis, and hypothesized that it may play a role in attenuating oxidative stress and anti-inflammation in COPD. Here, we validated the up-regulated SUMO2 expression and co-expressed lncRNA NONMMUT008873 in the OVA-induced asthmatic mice. Therefore, we speculate that Sumo2 may also play a role in asthma, and lncRNA NONMMUT008873 may play regulatory role in inflammation and oxidative stress by positively regulating transcriptional level of *Sumo2* mRNA during asthma.

Signal transducer and activator of transcription (STAT) pathway is a well-known signaling pathway associated with asthma [20]. STAT family contains seven proteins, and among them, STAT5 acts as a transcriptional activator in the immune response [20]. STAT5 consists of two isoforms, *STAT5a* and *STAT5b*, which can regulate lymphocyte proliferation, apoptosis, asthma and cancer [21]. Our results showed that *STAT5a* was decreased in the asthma model of mice, and this is supported by the another published research that the expression of *STAT3* and *STAT5a* genes in the peripheral blood mononuclear lymphocytes were down-regulated in the severe refractory asthma [22]. We also demonstrated that lncRNA NONMMUT009478 was decreased in the lungs of model mice, and had positive regulation on the *STAT5a*. Hence, the results may provide another regulatory target in asthma for further research.

Previous studies have found an increased expression of Fos in the peripheral blood mononuclear cells, monocytes and T cells from corticosteroid resistant asthma patients [23], and in the various brains regions of asthmatic mice [24]. Furthermore, Fos can be induced by Thymic stromal lymphopoietin and inhibited by Dexamethasone in the peripheral blood mononuclear cell of the patients with severe asthma [25]. Zhang et al. [26] identified that Fos was involved in the PI3K-AKT signaling pathway in patients with colorectal cancer using PPCR array. Liu et al. [27] reported that Fos was downstream of tumor necrosis factor in the KEGG pathway analysis, and played an essential role in cancer migration, proliferation, and invasion; furthermore, Fos was regulated by lncRNA RUNX1-IT1 and RUNX1 in the pancreatic cancer. Consistently, our microarray results showed that Fos and its lncRNA NONMMUT042325 expression was up-regulated in the lungs of asthmatic mice. Hence, inhibition of lncRNA NONMMUT042325 expression may have an important role in regulating the allergic inflammatory asthma.

SMG7 is a nonsense-mediated decay complex protein, and is responsible for the surveillance and degradation of abnormal RNAs [28]. SMG7 may directly interact with p53, regulate p53 stability, and lead to p53-mediated DNA damage response [29]. Yang et al. [30] suggested that SMG7 can protect against TNF-α-induced human cancer cell line apoptosis by regulating Pvt1 and the tumor suppressor CYLD. Our data showed that the gene expression and protein level of SMG7 and co-expressed lncRNA NONMMUT032848 was reduced in the asthma model of mice. These findings may provide new clues for identifying the therapeutic targets of asthma.

Additionally, we found an increased gene expression and protein levels of MYL9 in the OVA-induced asthmatic mice. It has been demonstrated that MYL9 can regulate ASMCs contraction through self-phosphorylation [31]. Xu et al. [32] reported that decrease of *Myl9* could attenuate ASMCs abnormal proliferation, migration, apoptosis and contraction, which could further lead to the airway remodeling in response to platelet derived growth factor, a well-known mediator to induce ASMC remodeling. We also found that lncRNA NONMMUT052633 had positive correlation with *Myl9*, which may be considered as a promising target for the treatment of asthma.

TLR4 is a well-known innate immune regulator and can participate in the development of asthma via interacting with TLR-related genes, such as high mobility group box 1 [33]. Shang et al. [32] suggested that TLR4/MyD88/NF-κB signaling pathway was closely related to asthma pathogenesis. Our microarray data showed that two lncRNAs could regulate the expression of *Tlr4*. NONMMUT053402 had positive correlation with *Tlr4*; on the contrary, lncRNA NONMMUT006807 had negative correlation with *Tlr4*. The expressions of these genes were all validated by qRT-PCR. Therefore, the lncRNAs of NONMMUT053402 and NONMMUT006807 may have functions related to the allergic inflammatory states of asthma.

Besides OVA, HDM, as the most common inhaled human allergen [34], is also commonly used for establishing allergic asthma model in mice [14, 35]. Compared with OVA-induced asthma model, HDM induced asthma model is more responsive to human allergen. HDM can disrupt the tight junctions in the airway, induce the production of cytokines, chemokines and collagens, and enhance the degranulation of eosinophils and mast cells [35]. Our laboratory is now working on the establishment of HDM-induced asthmatic mouse model. We will further conduct comparative studies on OVA- and HDM-induced asthma models.

## Conclusion

In summary, our microarray data revealed that when there was allergic airway inflammation, expressions of multiple genes were altered. The core mRNAs and co-expressed lncRNAs were screened out and further validated by RT-PCR and Western blot. The interactions of SMG7-NONMMUT032848, SUMO2-NONMMUT008873, STAT5a-NONMMUT009478, MYL9-NONMMUT052633, Fos-NONMMUT042325 and TLR4-NONMMUT006807/NONMMUT053402 were found to be involved in the allergic inflammatory asthma. Our findings may provide evidence for the development of therapeutic agents for bronchial asthma.

## Supplementary Information

**Additional file 1. Figure S1.** The un-cropped Western Blot images for Figure 5C, which showed the expression levels of Fos, Myl9, Smg7, Stat5a, Sumo2, tlr4 and β-actin in lung tissues of asthmatic mice.

## Data Availability

The microarray data presented in the study are publicly available and can be found at: https://www.ncbi.nlm.nih.gov/geo/query/acc.cgi?acc=GSE197090.

## Abbreviations

OVA: Ovalbumin
AHR: Airway hyperreactivity
ASMC: Airway smooth muscle cell
lncRNAs: Long non-coding RNAs
GO: Gene ontology
KEGG: Kyoto Encyclopedia of Genes and Genomes
