# Supplementary material for: Gene expression profiles and bioinformatics analysis in lung samples from ovalbumin-induced asthmatic mice
Source: BMC Pulm Med. 2023 Feb 2;23:50. doi: 10.1186/s12890-023-02306-w (PMC9893693; doi:10.1186/s12890-023-02306-w)
Supplement: Supplementary file 1 — Additional file 1. Figure S1. The un-cropped Western Blot images for Figure 5C, which showed the expression levels of Fos, Myl9, Smg7, Stat5a, Sumo2, tlr4 and β-actin in lung tissues of asthmatic mice. [file 12890_2023_2306_MOESM1_ESM.pptx]

## Slide 1
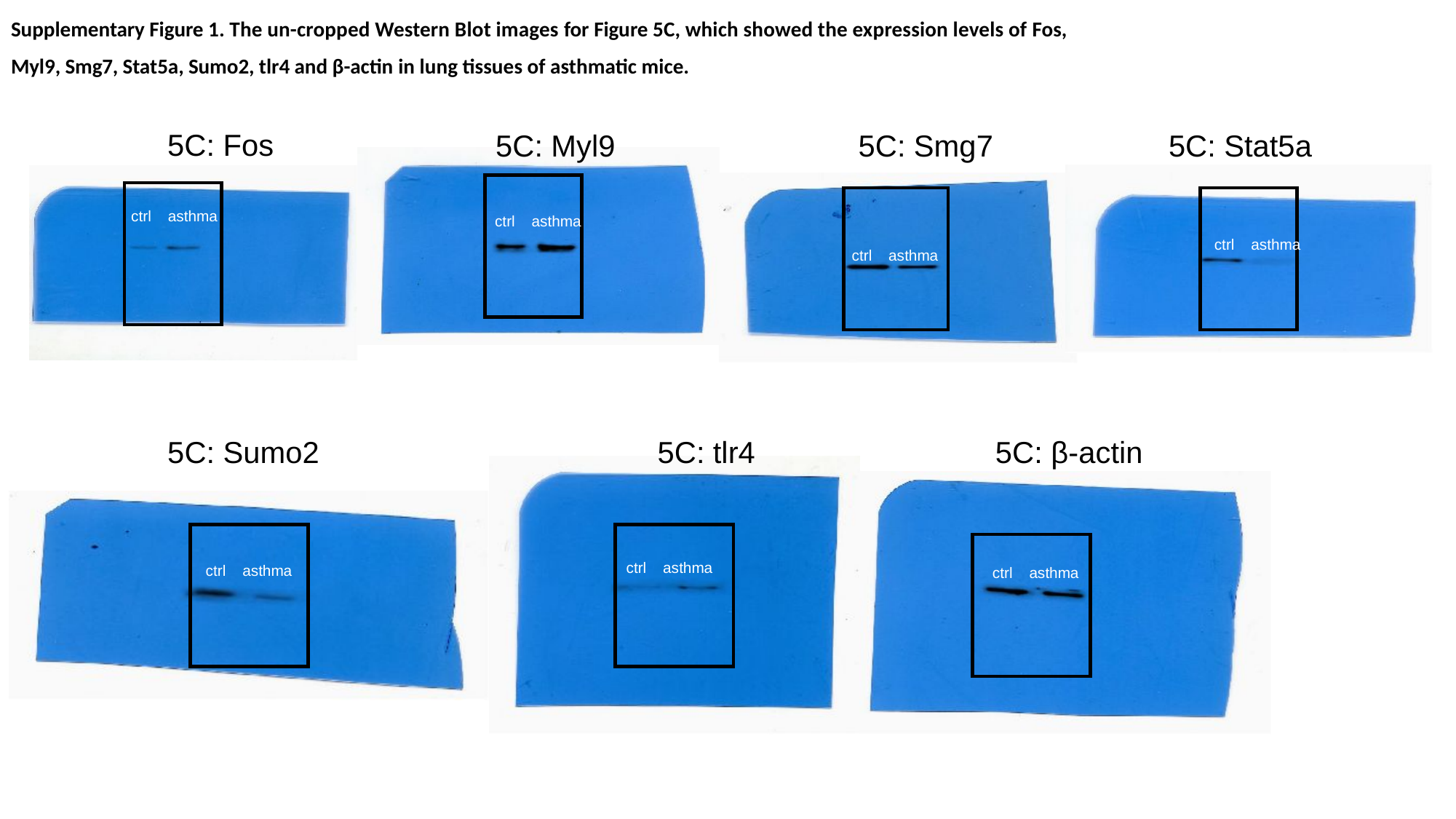

Supplementary Figure 1. The un-cropped Western Blot images for Figure 5C, which showed the expression levels of Fos, Myl9, Smg7, Stat5a, Sumo2, tlr4 and β-actin in lung tissues of asthmatic mice.
5C: Fos
5C: Myl9
5C: Smg7
5C: Stat5a
ctrl asthma
ctrl asthma
ctrl asthma
ctrl asthma
5C: Sumo2
5C: tlr4
5C: β-actin
ctrl asthma
ctrl asthma
ctrl asthma
